# Upadacitinib for treatment of recalcitrant central centrifugal cicatricial alopecia

**DOI:** 10.1016/j.jdcr.2025.12.019

**Published:** 2025-12-17

**Authors:** Christina Tolete, Adam Friedman, Joaquin Calderon, Leonardo Tjahjono

**Affiliations:** Department of Dermatology, George Washington University, School of Medicine and Health Sciences, Washington, District of Columbia

**Keywords:** central centrifugal cicatricial alopecia, JAK-1, scarring alopecia, Upadacitinib

## Introduction

Central centrifugal cicatricial alopecia (CCCA) is a progressive, inflammatory, cicatrizing scalp disorder that disproportionately affects women.^1^ CCCA is typically managed with a combination of systemic and topical and intralesional agents. Systemic options commonly include doxycycline, hydroxychloroquine, 5-α reductase inhibitors, and oral minoxidil. Topical corticosteroids and topical calcineurin inhibitors are typically used adjunctively.^2^ However, these treatments have limited reported efficacy. We report the first known case of recalcitrant CCCA successfully treated with upadacitinib.

## Case presentation

**Fig 1:**
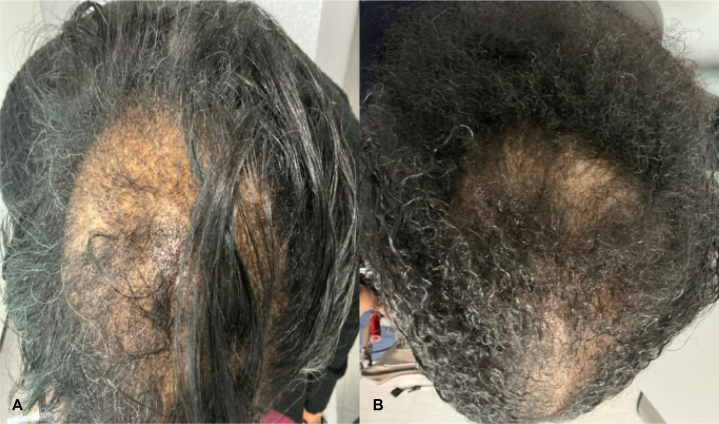
Clinical improvement in central centrifugal cicatricial alopecia with upadacitinib therapy. **A,** Baseline image showing vertex scalp alopecia. **B,** Three months after initiating upadacitinib, demonstrating visible hair regrowth.

**Fig 2:**
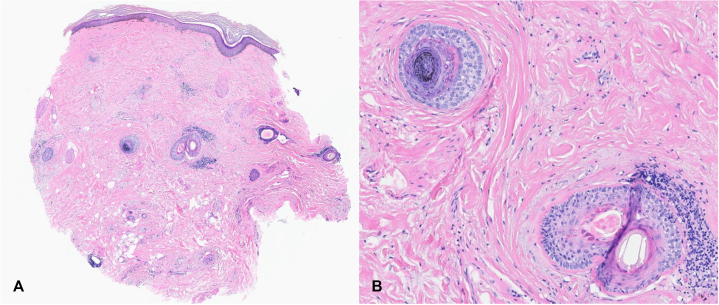
Histopathology of the scalp demonstrating perifollicular fibrosis, lymphocytic inflammation, and premature desquamation of the inner root sheath, consistent with central centrifugal cicatricial alopecia **(A)** hematoxylin–eosin stain, original magnification × 4 and **(B)** hematoxylin–eosin stain, original magnification × 20.

A 66-year-old female with no significant gynecology history presented in July 2024 with complaints of tender, progressive hair loss, previously managed only with oral minoxidil 2.5 mg daily for 1 year. Her mother also exhibited the same pattern of undiagnosed hair loss. She denied a history of tight hairstyles or recent significant stressors, such as surgery, infection, or social concern. She underwent menopause in her early 50s. Physical examination showed scarring alopecia most pronounced on the vertex and crown, with mild lipedema and significant scalp tenderness to palpation (Fig 1, *A*). Trichoscopy revealed perifollicular erythema and white halos. Punch biopsy on the vertex scalp demonstrated histopathological features supportive of a diagnosis of CCCA, with moderate scarring (Fig 2, *A* and *B*).

Initial therapy included doxycycline 100 mg twice daily, topical metformin 15% daily, hydroxychloroquine 200 mg twice daily, and a series of intralesional kenalog 5 mg/mL injections administered every 4 weeks, while continuing oral minoxidil 2.5 mg daily. After 3 months, however, scalp tenderness persisted, and the hair loss continued to progress.

Given the refractory and progressive course, treatment was transitioned to upadacitinib 15 mg daily, with discontinuation of doxycycline, hydroxychloroquine and intralesional kenalog; oral minoxidil and topical metformin were maintained. At 6-m follow-up, scalp tenderness had subsided, and noticeable hair growth was observed on the frontal and vertex scalp (Fig 1, *B*). No adverse events were reported, and the regimen was continued.

## Discussion

To our knowledge, this is the first documented case of CCCA successfully treated with upadacitinib, establishing it as an additional off-label therapeutic option for this recalcitrant condition. While its pathogenesis is incompletely understood, evidence suggests involvement of the Janus kinase (JAK)-signal transducer and activator of transcript pathway, which recruits T helper 17 cells that drive inflammation and follicular scarring.^3^

Further supporting this mechanism, brepocitinib - an oral JAK1/tyrosine kinase2 inhibitor with similar activity as upadacitinib - has shown efficacy and tolerability in CCCA. It also downregulates CCL5, a cytokine implicated in disease pathogenesis.^4^ Although multiple reports describe upadacitinib in lichen planopilaris, this is the first attempt to treat recalcitrant CCCA with updacitinib.^5^

CCCA can overlap with other hair-loss disorders such as traction alopecia, androgenetic alopecia, or telogen effluvium, which may have contributed to the clinical presentation. This overlap also makes it challenging to fully determine the relative impact of prior therapies, including oral minoxidil, which may have improved any component of nonscarring alopecia. However, given the rapidly progressive nature of the patient’s disease and symptom recalcitrance despite multitude of therapies and stable dose of oral minoxidil for 1 year, upadacitinib appears to have played the primary role in the observed improvement. In our patient, tenderness resolved and hair regrowth was observed after initiation of upadacitinib, with the most notable improvement occurring along the vertex and frontal scalp, despite prior progression on oral minoxidil and multiple immunomodulators. While minoxidil was continued, the stagnation in progress and stability of her regimen prior to JAK inhibition suggests that upadacitinib was the primary driver of symptoms and clinical improvement. Oral minoxidil and oral upadacitinib target distinct pathogenic pathways in CCCA and may be used concurrently to address different aspects of disease activity. Although this may represent a promising option for a devastating diagnosis with limited reliable treatment options, larger randomized controlled trials should be conducted to better assess its efficacy and safety.

## Conflicts of interest

Dr Tjahjono has served as a consultant and/or speaker for Arcutis, Bristol Myers Squibb, Eli Lilly, Incyte, Leo pharma, and Galderma. Dr Friedman is on the consulting/ad board of La Roche Posay, Galderma, Kenvue, Microcures, Leo Pharma, Pfizer, Hoth Therapeutics, Zylo Therapeutics, Mino Labs, J&J, Arcutis, Lilly, UCB, Novartis, UCB, Regeneron/Sanofi, Takeda; is a speaker for Regeneron/Sanofi, J&J, Incyte, UCB, Galderma, Arcutis, Lilly, Pfizer, and Novartis; received grants from Pfizer, Lilly, Galderma, Incyte, J&J, Abbvie, Loreal, and Regeneron/Sanofi. Dr Calderon and Author Tolete have no conflicts of interest to declare.
